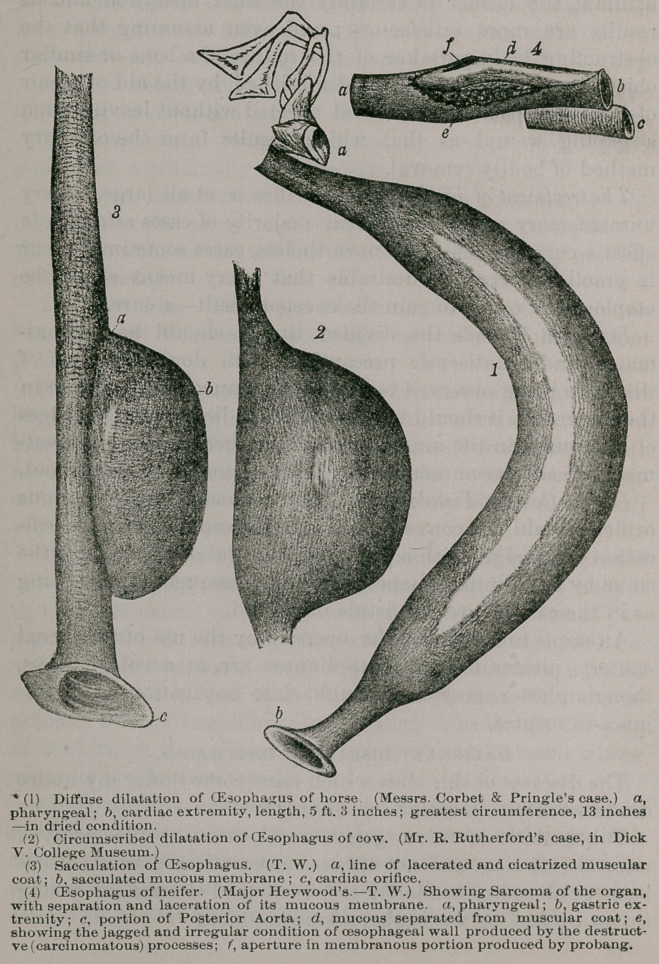# Organic Diseases of the Œsophagus

**Published:** 1887-04

**Authors:** Thomas Walley

**Affiliations:** Principal Royal Veterinary College, Edinburgh


### THE JOURNAL

OF

### COMPARATIVE MEDICINE AND SURGERY.

VOL. VIII. APRIL, 1887. No. 2.

### ORIGINAL COMMUNICATIONS.

### ART. IX. ORGANIC DISEASES OF THE ŒSOPHAGUS

BY THOMAS WALLEY, F. R. C. V. S.,
*Principal Royal Veterinary College, Edinburgh.*

In considering the organic diseases of that most important organ, the œsophagus, it is necessary to devote a brief space to the consideration of its *functional derangements.* Fortunately these are only two, viz: *loss of power* and *excess of power.*

(1) *Loss of Power.*—Paralysis of œsophagus, or œsophageal akinesis, may be *temporary* or *permanent;* the former resulting usually from exhaustion in attempts to propel onwards an abnormal collection of ingesta or to regurgitate an unusually large cud; to muscular injury, or to temporary nervous paralysis; the latter being associated with and due to important organic changes in the nerves or the muscular tissue.

Muscular injury, producing temporary paralysis, is usually inflicted in the act of swallowing sharp and unyielding objects; by the exercise of unnecessary violence in the passing of the probang; by the malicious introduction of sticks into the œsophagus (the pharynx usually being involved, in these cases); and by injuries inflicted from without.

Nerve derangement may be the result of some central or peripheral derangement of the tenth nerve, or to actual section of that nerve in such operations as phlebotomy.

It is manifest that a prolongation or extension of the above mentioned causes, will, in the long run, produce permanent paralysis; but there are frequently other influences, both nervous and muscular, in operation to produce this result. The nervous influences, are, undoubtedly, paralysis of the pneumogastric nerve, whether proceeding from central (brain) lesions or from peripheral influences, and atrophy of that nerve. The muscular influences are, atony of the muscular fibres, as the result of inherent imperfections of nutrition or imperfect nerve-supply; and atrophy produced by fatty degeneration consequent upon inflammation.

While recognizing, theoretically, the above causes as being in operation to produce œsophageal paralysis, it must be acknowledged that it is sometimes difficult to reconcile theory with clinical facts. It is a recognized law, that when nerve-force is withdrawn from muscular structure the latter undergoes important organic changes in the direction of fatty transformation and atrophy, and yet we occasionally meet with instances (as in Corbet and Pringle's case, hereafter to be related) in which we have undoubted evidence of nerve-deficiency coupled with increased muscular development, and associated with œsophageal akinesis.

Œsophageal paralysis, if persistent, is inevitably accompanied by œsophageal dysphagia, and by a marked tendency to impaction, followed by dilatation of the tube, and that, usually, along its whole length. While dysphagia is a marked symptom, there is not, except in the case of actual wounds, any evidence of pain, either on the application of external pressure, by the introduction of the probang, or in attempts to swallow; neither is spasm produced by any of these influences.

*Therapeutic Indications.*—If the paralysis is due to temporary causes, it will be succeeded by restoration of function on the removal or subsidence thereof; consequently, active treatment is not called for; regulation of diet with the avoidance of any irritating influences is all that is demanded. If, however, good grounds exist for the supposition that organic nerve or muscular change is in progress, more active therapeutic measures are required. These consist in the use both externally and internally of nerve and nervo-muscular stimulants. Of the former, no agent is comparable with strychnine (though physostigmine may be employed in the more recent cases where there is a tendency to impaction), of the latter, the application of the Actual Cautery over the Cervical portion of the œsophagus, or the employment of galvanism are the most effectual. Stimulating embrocations and even blisters may be tried in the early stages.

In all cases, due attention must be paid to feeding and watering, and on no account should the animal be allowed to get hungry or thirsty, as in its endeavors to satisfy the cravings of hunger and thirst, choking (impaction) is frequently produced and the existing lesion thereby materially aggravated.

Spasm (Exalted Function, Hyperkinesis) of œsophagus, "*Œsophagismus*," "*Dysphagia Spastica.*" Spasmodic contraction of the muscular walls of the œsophagus may be excited in any animal; it is most common and most easily produced in the horse. As an accompaniment of hysteria it is of common occurrence in the human female, in whom, owing to a sensation of a ball in the throat, it constitutes "Globus Hystericus," and from the fact of its producing temporary occlusion it has been designated *temporary, or spasmodic stricture.*

*Causes.*—While spasm is usually excited by the lodgment of foreign matter, as in the condition known as choking, and is a necessary accompaniment of vomiting and eructation, it not infrequently arises in the horse without the intervention of any such apparent cause, and can only be attributed to some peripheral or central irritation of the pneumogastric nerves. *Symptoms* are always most pronounced, and often distressing, in the horse.

The spasm when of idiopathic origin, is intermittent, and though usually excited by the act of swallowing, especially cold water, it not infrequently occurs while the animal is quiescent. On the advent of the spasmodic contraction, the nose of the sufferer is suddenly protruded, the neck is drawn downwards and backwards, and sometimes curved laterally; the ears are fixed, either in a backward or forward direction, and the œsophagus is observed, on the left side of the neck, to shorten itself and to roll up in the form of a hard or unyielding mass. In the horse, a scream may be emitted. There is, usually, no cough, neither is there any pain evinced on the application of pressure; there is no salivation or constitutional disturbance. If the spasm persists, it may lead to the production of organic disease.

*Treatment.*—Hand rubbing and hot fomentations, followed by the application of stimulating anodyne liniment. Belladonna or subcutaneous injections of atropine, conia, or morphia, with the internal administration of one or more of these agents. Hard or irritating foods should be avoided as should, also, very cold water; and the animal should be fed and watered sparingly but often.

### INFLAMMATION OF THE ŒSOPHAGUS.

*Œsophagitis* is seldom, or never seen in the domestic animals as an idiopathic affection.

*Traumatically,* it is produced by lacerations inflicted by the passage of sharp or hard roots or foreign bodies; by violence in the use of the probang, by the malicious thrusting of sticks down the throat, and by external injuries—in all these cases the inflammation is circumscribed.

*Acute Catarrhal Inflammation,* is frequently produced by the action of corrosive agents, as strong acids and alkalies and very hot water—nitrate of potash is probably the only agent of a non-corrosive character capable of inducing acute œsophagitis.

Catarrhal croupous or a diphtheritic inflammation occasionally occur as an extension from the fauces and pharynx, and some of the worst cases of the two latter forms I have ever seen have occurred in the ox and the pig. Catarrhal sometimes results from extension from the stomach.

*Interstitial Inflammation,* may be the result of extension from the mucous membrane in mucositis; more largely it is induced by injury.

The cardiac extremity of the œsophagus is, in the horse, frequently (much more so than is imagined by most practitioners) the seat of interstitial changes, the walls of the tube in many instances becoming enormously thickened for a distance of several inches and its lumen diminished to the diameter of a crow's quill. It is undoubtedly secondary, in many cases, to interstitial gastritis and arises as an extension thereof.

*Indications* are, Œsophageal Dysphagia, indeed, indifference to the taking of food is usually the first symptom manifested; pain is evinced on the application of external pressure and on the administration of irritant medicines. Tumefaction of the organ may or may not be present; the nose is usually protruded in order to remove the pressure. Diagnosis is sometimes facilitated by the history of the case, and where the lesion is suspected, it should be carefully enquired into.

*The Therapeutic Indications* are, to prevent gastro-intestinal complications by the administration of laxative medicines; to reduce the inflammatory action as quickly as possible by the administration of sedatives—tincture of aconite and nitrate of potash *par excellence;* and by the external application of fomentations with belladonna or opium liniments, or arnica lotion; and after the subsidence of the inflammation, a counter irritant over the cervical portion of the organ. Careful dieting both during the existence of the process and subsequently, is imperatively demanded.

*Sequelæ.*—Œsophagitis, no matter how produced, is of greater gravity in its results than in its immediate effects, as it may act as an exciting cause to the development of such malignant affections as carcinoma, sarcoma and tuberculosis.

Catarrhal or other forms of œsophageal mucositis may be followed by ulceration which in its turn may produce by contraction in the healing process, stricture. Acute inflammation may, and sometimes does, induce fatty changes in the muscular walls thus predisposing to dysphagia and dilatation; while interstitial inflammation is a frequent cause of constriction.

### ABSCESS

May be a result of acute phlegmonous inflammation; it is more largely produced, however, by direct mechanical injury, and when arising in the thoracic portion of the tube is a very grave lesion as its contents may be evacuated into the thorax, in which case, acute pleurisy and death inevitably result. In the cervical region the contents of the abscess may be evacuated externally, and if suppuration has resulted from direct injury from within, as from the penetration of a foreign body or from a wound inflicted by the probang, it will certainly be followed by œsophageal fistula. Evacuation of the contents of the abscess into the lumen of the tube is the most desirable termination, but even in the event of this taking place, sacculation—or one form of it—is to be dreaded. The signs of the formation of œsophageal abscess are those of acute disease of the tube from any cause, and if situated in the cervical region the usual indications of the process are manifested externally.

*Therapeutic indications* are similar to those of œsophagitis; in all cases where the abscess is situated in the cervical section of the tube the pus should be evacuated, surgically, immediately its presence is diagnosed; and the use of the exploring needle will enable the practitioner to do this at a tolerably early period of its formation. The longer pus is imprisoned, the greater will be the destruction of tissue and the greater the probability that grave sequelæ as stricture, sacculation, or fistula will arise.

RUPTURE AND LACERATION OF THE ŒSOPHAGUS.

Rupture may be *partial* or *complete*; it can scarcely be produced by such indirect violence as vomiting, so long as the organ is structurally healthy, nor does the impaction of foreign bodies lead to such a result under ordinary circumstances. The lesion is, as a rule, produced by violence in the removal of foreign bodies, especially where these are of an angular formation or present salient or sharp points of projection. Laceration from without may occur in any animal, but wounds inflicted in the above mentioned manner are most frequently met with in cattle. Very exceptionally, rupture is produced by the spasmodic contraction of the œsophagus upon the probang, in its withdrawal, when that instrument is used for the displacement of foreign bodies. The indications of the occurrence of rupture during the act of dislodging foreign bodies are, shrinking of the animal—probably groaning or, in cattle, bellowing—when force is applied with the probang, and on the withdrawal of the instrument it is usually found smeared with blood.

The subsequent general indications are, inappetence, arrest of rumination in ruminants; gastric tympany, great prostration and depression; salivation, moaning and gnashing of the teeth, in cattle and sheep, with rapid and irritable pulse and more or less febrile disturbance.

The local indications are, in the cervical region—heat, tenderness and swelling along the course of the œsophagus with (especially in cattle) diffuse emphysema, this condition being produced by the infiltration of gas regurgitated from the stomach. If the foreign body has been forced through the tube, it may, in the cervical region be detected under the skin by manipulation. If the injury is located in the thoracic section of the tube, chest symptoms of obscure character will be manifested. In either case the cautious passage of the probang will determine the existence and site of the injury, signs of pain being immediately evidenced when the extremity of the instrument comes in contact with the damaged tissues.

*Surgical Indications.*—If the injury is localized in the neck and the foreign body can be detected it should be removed by means of an external incision with as little delay as possible, and the injured œsophagus freely exposed; after the removal of the object, all foreign matter should be carefully removed and the edges of the wound closely approximated with aseptisized silk sutures. No solid food of any kind should be allowed for a period of ten or twelve days, and even then very cautiously and when there is reason to suppose that the wound has healed a small probang should be passed two or three times a day; subsequently probangs of a gradually increased size should be used—by this means stricture may be avoided.

If the injury is located in the chest, palliative treatment can alone be adopted. Practically nothing more than the exercise of great care in dieting can be done.

*Results.*—Solution of continuity of the walls of the œsophagus, even though of slight extent, is always a grave lesion. If partial, ingesta will become insinuated between the coats of the organ, and, acting as an irritant, set up acute inflammation followed by suppuration and the formation of an abscess; or it will lead to the production of stricture.

If the rupture is complete, the foreign body escapes into the tissues surrounding the tube, and in its thoracic portion may pass into the chest, or if situated at that portion of the œsophagus contacting with the root of the lungs, it may be lodged in the connective and mediastinal tissue of the part and be followed by an abscess. If thoracic rupture is complete, ingesta and water find their way into the chest and establish a fatal pleurisy.

FISTULA OF ŒSOPHAGUS

Is, fortunately, seldom seen in veterinary practice, and more frequently in cattle than in any other class of animals.

*Causes.*—1st. Perforating ulcer, no matter how originating.

2d. The destructive processes associated with and consequent upon such malignant diseases as cancer and sarcoma.

3d. Penetrating wounds inflicted by foreign bodies from within, or, accidentally or otherwise, from without.

4th. The formation of an abscess in close proximity to the œsophagus; the pressure exerted by it causing absorption of the walls of the tube and the contents subsequently gaining exit externally.

The worst case of this kind which has come under my notice occurred in a colt, under the care of Mr. Campbell, of Kirkcudbright.

5th. Rupture of the œsophagus in attempts to dislodge foreign bodies, the object being forced through the walls of the tube, there acting as an irritant, inducing the formation of an abscess, and being evacuated with the pus, or removed surgically. A case of this kind came under my observation many years ago, while a pupil with Mr. Kettle.

6th. Fistula is one of the most unpleasant and most dreaded results of œsophagotomy, inasmuch as a wound in the œsophagus is kept patent for an indefinite period by the constant passage of solid or fluid matter when attempts are made to swallow.

Fate, probably, has no more miserable existence in store for an unfortunate animal than that of œsophageal fistula; perpetually hungry ingestion cannot satisfy it, as the greater part of that which it swallows does not reach the stomach but escapes by the fistulous opening.

I have already indicated that the majority of cases of œsophageal fistula result from the surgical removal of foreign bodies, this arises from the fact that attempts are usually made to remove these bodies *en masse*, instead of adopting the slower but safer method of removing them piecemeal. Given a tuber, *e g.*

potato, or a portion of a root, as of a turnip, it is surely better to make a small incision directly over it, and by the aid of a gouge break down the obstructing object and remove it in small particles than to boldly cut through the walls of the œsophagus and remove it bodily. The latter may be the more brilliant, the former is certainly the safer operation, and its results are more satisfactory; and even assuming that the obstructing body partakes of the nature of a bone or similar object, its dimensions can be diminished by the aid of a pair of bone forceps and its removal effected without leaving such a gaping wound as that which results from the ordinary method of bodily removal.

*The treatment of Fistula,* if the orifice is at all large, is very unsatisfactory and in the great majority of cases attempts to effect a cure are defeated; nevertheless, cases sometimes occur in practice where it is desirable that every means should be employed in order to gain the coveted result—a cure.

*In Recent Wounds* the divided tissues should be approximated, under antiseptic precautions, with double strands of silk, care being observed to include the mucous membrane in the suture, but it should not be allowed to lie between the edges of the wound in the muscular coat; in other respects the treatment already recommended for rupture should be carried out.

*In Old Cases of Fistula, with Callous Boundaries,* the fistulous orifice should be converted into a new wound by free scarification of its edges with a gouge, or removal of the walls of the sinus by an elliptical-shaped incision, subsequently proceeding as in the case of a recent fistula or wound.

Attempts to obliterate the opening by the use of the actual cautery plaster, or similar appliances, are, as a rule, failures, though plaster may be brought into requisition as an adjunct to sutures.

### MALIGNANT DISEASE OF ŒSOPHAGUS.

The diseases of this class which have come under my notice are tuberculosis, sarcoma and cancer.

Tuberculosis of this organ is rarely seen as the result of natural infection, and not often as a secondary disease; it has been produced, secondarily to pharyngeal disease, in young calves by feeding with tuberculous products.

The two latter conditions are more common, though, fortunately for the sufferers, they too are of comparatively rare occurrence. I have only seen one or two cases in the horse, of secondary origin to faucal and pharyngeal disease, and altogether four or five in cattle.

One of the worst cases, though it was more circumscribed than others I have seen, occurred many years ago, when I was practicing in North Wales, in a two years old heifer of the Montgomeryshire smoky-faced breed; and in this case the diseased processes led to so much attenuation of the mucous membrane as to cause it to yield to the pressure of a probang which I had passed with the view of assisting in the diagnosis. The conditions produced by the destructive processes in this case are depicted in Fig. 4.

Unless originating as an extension of disease from the pharynx, the mucous membrane withstands the destructive effects of malignant disease, such as cancer or sarcoma, for a considerable period.

THE INDICATIONS OF MALIGNANT DISEASE

are not always diagnostic; they are as follows: œsophygeal dysphagia, arrest of ingesta at a certain point, voracious or morbid appetite, marked gastro-intestinal derangement as shown by tympany (more or less persistent), irregularity of the bowels and frequent attempts at vomition; emaciation and general unthriftiness of the body, and death from inanition. In all suspected cases of this kind, the probang should be cautiously passed and the site of the disease localized; and in those cases in which the mucous membrane is engaged in the diseased process the extremity of the probang, on withdrawal, should be examined for evidence of debris; if any be present it should be microscopically examined in order to determine its character.

In the cervical region, external confirmatory evidence may be obtained by careful manipulation. *Treatment* of such cases is, from their nature, hopeless; palliative measures may be adopted, but even if the disease could be arrested, stricture would almost certainly result.

On microscopical examination, *post-mortem,* it will usually be found that the tissues around and adjacent to the œsophagus are involved in the destructive process; particularly is this the case with the bronchial lymphatic glands, when the disease is localized in their immediate proximity. I have in one or two instances thought that the disease had primarily originated in these organs.

STRICTURE OF ŒSOPHAGUS may be localized in any part of the tube; in the horse, as already indicated, its seat is usually in the cardiac extremity.

*The Causes.*—May be *intrinsic* or *extrinsic.* In the former class we have thickening of the œsophageal walls, as the result of inflammation—especially interstitial—abscess, malignant disease, contraction of the cicatrix in the healing process of wounds or ulcers, and the formation of polypi—the last mentioned being of very rare occurrence.

In the latter class of cases, we have the pressure exerted by external growths adjacent to the œsophagus, enlarged bronchial lymphatic glands, constriction by contraction of pleural adhesions in the thorax, invagination between the tracheal rings, and disease of the diaphragmatic structures around the foramen sinistrum.

Stricture may be *partial* or *complete.* It may, or may not, be associated with dilatation. In chronic cases this complication is inevitable as the repeated lodgment of ingesta at the point of obstruction causes the walls of the œsophagus to yield gradually to the eccentric pressure exerted upon them.

*The Indications of Stricture* depend upon the extent to which the lumen of the tube is occluded, and also upon the age of the lesion. They are œsophageal dysphagia with impaction, these conditions being most observable when the sufferer is allowed to eat or drink *ad lib.*, after a period of fasting.

It is of the utmost importance to distinguish between this lesion and pharyngeal obstruction, or dysphagia. In the former, the symptoms do not make their appearance until the food or water has reached the point of obstruction, and a great part of the œsophagus anterior thereto becomes distended with the solid or fluid matter. In the latter they appear immediately the animal endeavors to swallow. Glutting and rejection of food or water by the nostrils, in the horse (the mouth, and *probably* nostrils also, in other animals,) taking place at once, whereas in œsophageal stricture some little time elapses before pronounced symptoms are observed, and as these will be considered fully in speaking of dilatation, they need not be further considered here.

Whenever stricture is suspected its site and extent should be accurately localized by carefully passing the probang, and in doing this, recent may, as a rule, be readily differentiated from chronic, by the facility with which the introduction of the instrument is effected, and by the presence or absence of blood stains on the end of the probang when it is withdrawn. The difficulty of introducing the instrument is greatest in chronic cases; while in recent, blood stains are in the majority of instances observed.

Stricture, like fistula, leads ultimately to extreme emaciation and produces death by inanition.

*Indications as to Treatment.*—1st, to give only such foods as are easy of deglutition; 2d, to carefully regulate the dieting so as to avoid hunger; 3d, to endeavour to overcome the stricture by the judicious use of bougies of gradually increasing sizes. If there are indications of great interstitial thickening, potassic iodide may be administered internally, and if the stricture is localized in the cervical region, iodine may be applied immediately over its seat, or injected und r the skin.

DILATATION OF ŒSOPHAGUS.

*Œsophagus Ventricosus.*—Dilatation may be *diffuse*, or *circumscribed.* Diffuse dilatation may, and sometimes does, extend the whole length of the organ and its lumen may be enormously enlarged. In the œsophagus depicted in Fig. 1, the greatest circumference is, in its dried condition, 13 inches; the length 5 feet 3 inches. This specimen was forwarded to me by Messrs Corbett and Pringle, from New Castle-on-Tyne, some years ago; it was removed by them from the carcass of a 3-year old cart horse. Circumscribed dilatation does not usually involve more than a few inches of the tube; one of the worst cases I have seen, except in association with malignant disease, being that shown in Fig. 2, the specimen, removed from a cow, was placed in the collection of the College Museum by Mr. Richard Rutherford.

Dilatation may, and does take place independently of stricture (as in Messrs C. and P's case), but stricture cannot, as a rule, exist long without producing dilatation. A remarkable exception to this rule is seen in the case of stricture of the cardiac extremity (alluded to above) in the horse, and I have repeatedly seen this in dissection subjects and when making *post mortem* examinations.

Other complications of dilatation are: A. *Paralysis of the laryngeal muscles*; as in C. and P.'s case, the larynx and tenth nerve showing all the conditions usually observed in roarers. B. Organic disease as cancer, sarcoma, tuberculosis or ulceration.

The walls of the tube may be much attenuated and pale in color; on the contrary the muscular coat may be greatly hypertrophied and of a very florid hue—such was the case in the œsophagus depicted in Fig. 1. When the hypertrophy is due to the formation of new interstitial matter, the organ is pale in color. Organic changes in the mucous membrane are seldom seen in simple dilatation.

*The causes* of simple dilatation are: 1st. Atrophy of the muscular wall with or without fatty degeneration as a sequel of inflammation; or deprivation of nerve force as a result of injury to, or paralysis of, the pneumogastric nerve. 2d. Atony and attenuation of the muscular wall.

Dilatation resulting from stricture is due to continual accumulation of food in front of the constricted portion of the tube.

*The Symptoms* of Simple Dilatation are, as a rule, much less pronounced than are those of dilatation with stricture; they are usually evidenced, in the case of the horse, when the animal is put in the stable after work (fasting) and allowed to drink water *ad lib.*, or to eat at will of bran mash or other similar food; and are excited by the temporary *impaction* of food produced by inability on the part of the weakened œsophagus to force it onward. They are best observed when the cervical portion is engaged in the lesion, the animal showing the symptoms already described in speaking of spasm, and on examining the left side of the neck the distension is at once detected, as are also the movements in the tube, produced by the spasmodic efforts of the muscular coat to propel the contents towards the stomach.

If water is arrested in its progress, fluctuation can be distinctly felt, and on auscultation a gurgling sound is heard; this sound assists the practitioner to detect the condition in the thoracic portion of the œsophagus.

When water alone has been swallowed the symptoms pass off in the course of a few minutes, and the same holds good in the case of temporary lodgment of succulent mashes, but if the animal is very hungry and continues to devour voraciously such food as chaff, half or moistened bran or grass, the *impaction* becomes so great as to induce symptoms of extreme distress—particularly in the horse. The symptoms usually presented under these circumstances in the horse are salivation —often very profuse; paroxysmal spasm associated with squealing; sometimes lying down and rising again immediately, or going down partially or wholly on knees; perspiration more or less profuse; oppressed pulse, throbbing of the carotid arteries and turgescence of the superficial veins notably of the jugulars; great difficulty in breathing, accompanied by cough, usually paroxysmal in character; regurgitation of ingesta through the nostrils and eructation of gas and if the impacted matter has lain for long in the tube the ejected matter is alkaline in re-action instead of acid as in the case of vomition. Tympany sometimes slight, at others so pronounced as to cause great distress and threatened suffocation.

If relief is not afforded, death may ensue from exhaustion, or from asphyxia. The latter may be produced by the pressure of the gas in the distended bowels; by the pressure of the impacted matter on the trachea; or, by the passing solid or fluid material into the trachea and bronchial tubes. In Messrs. Corbet and Pringle's case, the œsophagus was simply packed with fresh grass, and a tolerable quantity of grass and water was found in the trachea and bronchia. This animal had been purchased at Newcastle Fair on the day prior to his death, travelled some little distance into the country, and placed on luxuriant pasture for the night; he was found dead the following morning.

In cattle, the symptoms of dilatation, and of dilatation with impaction, are much less marked than in the horse, and any material that may be ejected is passed through the mouth as well as through the nostrils. The dog succeeds much more readily in expelling impacted matter, and consequently obtains relief more easily than do other animals.

In reference to the general condition of an animal suffering from dilatation, I have seen horses, even in the worst cases, looking the picture of health, and capable of doing their share of work without manifesting signs of distress or exhaustion; but in chronic cases we usually have emaciation and unthriftiness, and great debility, with marked irregularity of the bowels; and in milch cows, partial or total arrest of the secretion of milk.

In complicated dilatation, *e. g.*, when associated with stricture, the symptoms above described are materially aggravated, and their duration prolonged.

*Treatment of Simple Dilatation.*—In no case should an animal the subject of this lesion, be allowed to go for any length of time without food or water; in the stable, in fact, the horse should have a permanent supply of water, and food should be given in small quantities and often. Hay is sometimes swallowed with greater facility than are softer and more succulent foods.

If the dilatation seems to be due to nerve paralysis, nerve stimulants should be administered and a strong stimulant, or even the flat-iron applied along the whole course of the cervical section of the tube. Strychnia may be injected subcutaneously, or galvanism may be had recourse to. In dilatation with stricture it is obvious that no permanent good can be effected by treatment unless the stricture is overcome.

When impaction takes place, all available means should be employed to remove the accumulated matter with as little delay as possible. The longer the condition continues, the firmer the mass becomes, and the greater is the difficulty experienced in dislodging it.

If ejection of portions of the impacted matter is being effected by the animal itself, the process should be encouraged by the careful administration of linseed oil or other lubricant, and by breaking the mass down, when the impaction is localized in the cervical region, by careful kneading applied externally. If the lodged matter is of a fluid character, it may be withdrawn by the aid of an aspirator or the trocar and canula; if semi-solid, water may be injected directly into the mass by an irrigating syringe. In no case should the probang be brought into requisition in dealing with solid or semi-solid material, unless the bulb is of small dimensions, as attempts to dislodge the mass by the aid of an ordinary probang only lead to its firmer impaction.

If tympany is so great as to cause distress, and interferes with respiration, it should be at once relieved by paracentesis, and if congestion of the lungs with oppressed action of the heart exist, mustard plasters or ammonia liniment should be applied to the chest, and blood, in moderately large quantity, abstracted from the jugular vein.

If the above measures fail in affording relief, œsophagotomy (when the impaction is in the cervical portion of the tube) should be performed as a *dernier resort*, but instead of cutting boldly down into the mass by a large incision, a small opening only should be made, and the mass dislodged by the aid of water injected with a double action syphon or syringe, assisted by a small blunt hook. In very bad cases an opening may be made at either extremity of the impacted mass and water injected from above downwards. Any subsequent contraction in the healing of the wounds will be useful. The plan here recommended may be put into execution in ordinary cases of impaction of the œsophagus with semi-solid or solid matter. Tracheotomy should be performed where there is imminent danger of suffocation taking place and the seat of impaction permits of the operation.

### SACCULATION AND POUCHING OF THE ŒSOPHAGUS.

*Œsophageal Diverticulum.*—Sacculation differs from dilatation in the important fact that there is solution of continuity of the structure of one of the coats, and while pouching is strictly a form of sacculation, the term should properly be confined to those cases in which there is an aperture through both coats communicating with the lumen of the tube and also with an extra œsophageal pouch formed in the tissues around the organ.

Sacculation of the mucous membrane, *complete sacculation*, is usually caused by laceration of the muscular coat by the passage of unyielding bodies, as by extreme pressure exerted with the probang in the act of unchoking, the muscular, on account of its firmer texture, yielding before the mucous coat.

Sacculation of the muscular coat, *incomplete sacculation,* is usually caused by laceration of the mucous coat by sharp foreign bodies or improperly constructed probangs, and by destruction of the mucous coat by ulceration or the action of corrosive agents.

Pouching is usually caused by the formation of an abscess adjacent to the œsophagus, or by the destructive process of sarcoma or cancer, or by complete laceration of its coats; in abscess the coats are destroyed by the suppurative process and the contents of the abscess discharged into the tube, the walls of the abscess forming the pouch; in the latter case ingested matter finds its way into the surrounding tissues, acts as an irritant and produces suppuration, but ultimately a distinct sac or pouch is formed as a result of interstitial growth.

The aperture of communication in complete sacculation may be a mere slit; on the contrary, it may be a wide open mouth, and the sac itself may become so large as to form a kind of cæcal appendage to the œsophagus. This condition is well shown in Fig. 3.

No matter how originating, the size of the sac or pouch, owing to the constant passing into it of ingested matters, is liable to increase, and it will always be largest in those parts where the least support is afforded by surrounding tissues or organs.

One of the most remarkable cases of pouching that it has been my lot to witness occurred in the practice of Mr. Walters, of Newport, Salop.

The subject was a cow, and the case came under the notice of Mr. Bampfield Kettle, of Market Drayton, while acting as *locum tenens* for Mr. Walters. The site of the pouching was opposite the root of the lungs, and the pouch was of at least two quarts capacity. It bore evidence of having been in existence for a considerable period, and it had evidently acted the part of a diverticulum, and a constant interchange of ingesta had been going on between it and the œsophagus.

*The Indications of Sacculation and Pouching* are tolerably distinct when the lesion is situated in the cervical portion of the œsophagus, but very indefinite when localized in the thoracic portion. In the former case, an intermittent tumor, or globular shaped swelling, will be readily detected by the eye and hand, its consistence and character varying with the character of its contents, *i. e.*, whether fluid, semi-fluid or solid. Except in the early stages of its formation, or in the event of inflammatory action being set up from any cause, there is neither heat, pain, nor tenderness, and the general condition of the patient may be good. On the contrary, the animal may be unthrifty and emaciated. When situated in the thorax, the general symptoms are similar to those observed in dilatation with stricture, and differentiation of the two conditions is scarcely possible.

Impaction may take place in the same manner as in dilatation, but unlike that condition, there is a great probability of suppurative inflammation being induced by the prolonged retention of ingesta. The general indications as to treatment, are, on the whole, similar to those of dilatation, but when the lesion is localized in the neck, some benefit may accrue from the application of a compress and bandage, or from the application of strips of adhesive plaster crosswise over the sac. Alternatively, repeated blisters may be applied, or iodine injected subcutaneously with the object of establishing a formative inflammatory process, and by thus increasing the quantity of interstitial tissue round the sac, affording a certain amount of support to it. Care, however, in dieting and watering is of more importance than is the direct application of any particular method of active treatment.

*The legal aspect* of chronic œsophageal disorders may be dealt with in a few words.—Whenever it can be shown that any organic lesion of the œsophagus has been in existence prior to purchase, and the animal, be it of what species it may, was warranted sound, the warrantor is most certainly liable for damages, even though a considerable period may have elapsed after the date of sale.

## Figures and Tables

**Figure f1:**